# Bisacodyl micro-enema before MRI of rectal tumors: effects on rectum, image quality and patient acceptance

**DOI:** 10.1007/s00330-025-11996-1

**Published:** 2025-09-17

**Authors:** Ellen Viktil, Bettina Andrea Hanekamp, Arild Nesbakken, Ole Helmer Sjo, Anne Negård, Johann Baptist Dormagen, Anselm Schulz

**Affiliations:** 1 Department of Radiology, Oslo University Hospital Ullevål, Oslo, Norway; 2 Institution of Clinical Medicine, University of Oslo, Oslo, Norway; 3 Department of Gastrointestinal Surgery, Oslo University Hospital Ullevål, Oslo, Norway; 4 Department of Radiology, Akershus University Hospital, Lørenskog, Norway

**Keywords:** Rectal neoplasms, Magnetic resonance imaging, Enema, Image enhancement, Patient acceptance

## Abstract

**Objectives:**

To assess the effects of a bisacodyl micro-enema on rectal physio-morphology, image quality, and patient acceptance when used as preparation before MRI of rectal tumors.

**Materials and methods:**

In this prospective single-center study, we made an intra-individual comparison of patients with suspected early rectal cancer who all completed MRI without (MRex) and with (MRin) a bisacodyl micro-enema. The width and the anal-oral extension of the submucosal edema in the healthy rectal wall, anal-oral extension of luminal fluid, and rectal distension were registered. Image artifacts were recorded with a figurative visual analog scale (fVAS), and patient acceptance with questionnaires at baseline, after MRex, and MRin. Significance levels were calculated with a *t*-test, the Wilcoxon signed-rank test, or McNemar’s test for paired samples and inter-reader agreement with Gwets AC1 statistics.

**Results:**

Consecutively, 50 patients (mean age, 65 years ± 10, 26 men) were included. The median (iqr) summed width of submucosa (MRex, 26 (12) mm, MRin 39 (13) mm, *p* < 0.01) and anal-oral fluid extension (MRex 0 (0) mm, MRin 134 (91) mm, *p* < 0.001) increased. Susceptibility artifacts on diffusion weighted imaging (axial plane *p* ≤ 0.04 and parallel plane *p* < 0.001) were reduced. No increase in rectal distension, motion artifacts, or side effects was registered, and the patient-reported acceptance remained unchanged.

**Conclusion:**

Bisacodyl significantly increased the submucosal width and extension of intraluminal fluid, did not lead to distension of the rectum, improved image quality, and was well tolerated by the patients.

**Key Points:**

***Question*** *Bisacodyl micro-enema can improve MRI of early stage-rectal tumors, but the impact on rectal physio-morphology, image quality, and patient acceptance is unknown.*

***Result*** *Bisacodyl micro-enema increased the submucosal width and intraluminal fluid, did not distend the rectum, improved image quality, and was well tolerated by the patients.*

***Clinical relevance*** *Bisacodyl micro-enema represents a reliable preparation before MRI of rectal tumors and can improve imaging of early-stage cancer. This is increasingly important because of the shift toward earlier stages due to screening programs and the growing use of organ-saving treatment.*

**Graphical Abstract:**

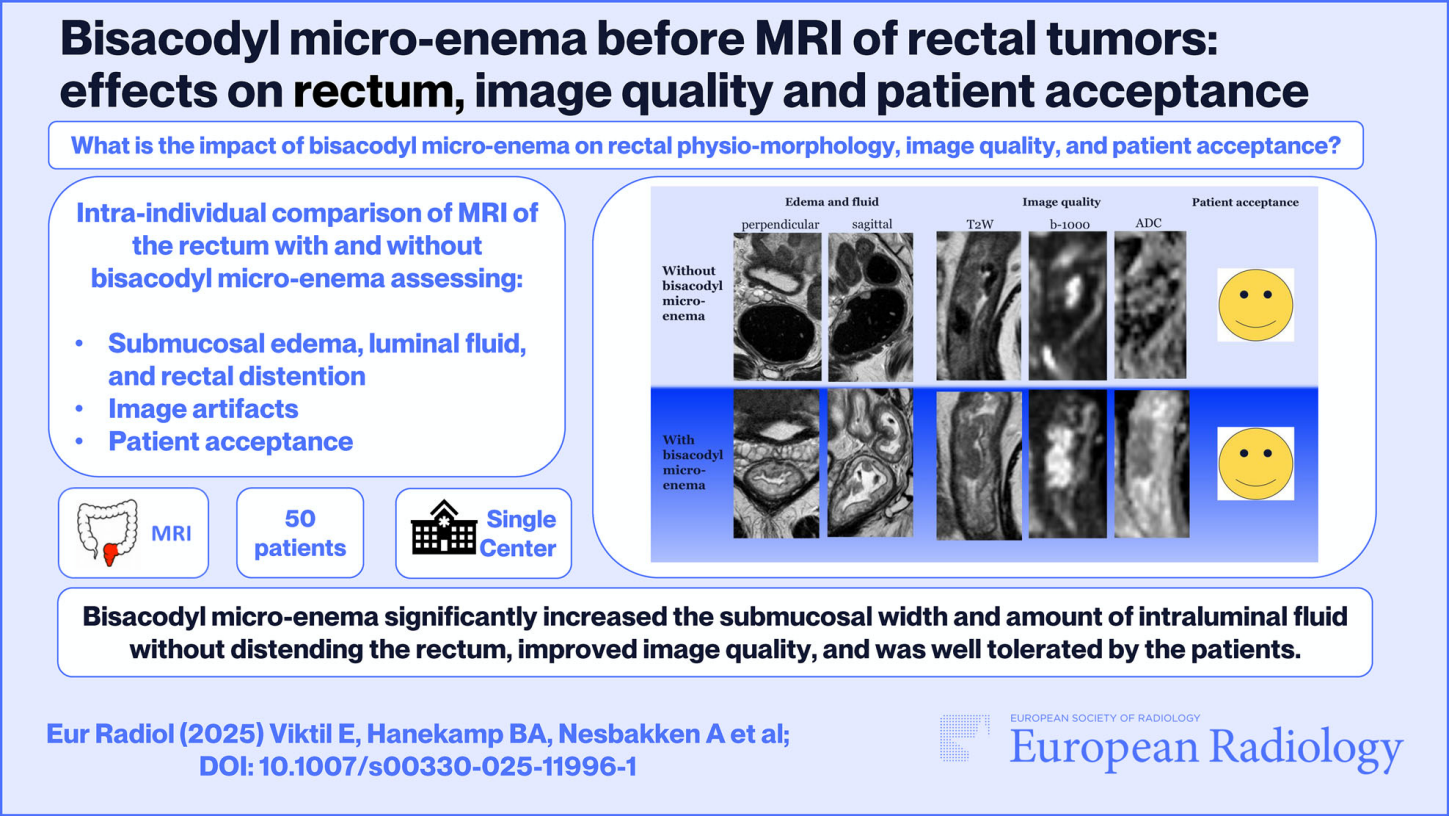

## Introduction

For many pelvic diseases, treatment decisions are based on MRI examinations, and high image quality is essential. Several patient preparation methods have been investigated to improve image quality, including dietary restrictions, rectal filling, micro-enemas, and antiperistaltic drugs.

No consensus exists on the use of rectal filling or micro-enemas before MRI of rectal cancers, and both measures are controversial and not routinely recommended in guidelines [1, 2]. Preparatory micro-enemas have been found advantageous for image quality on diffusion weighted imaging (DWI) in response evaluation of rectal tumors after chemoradiotherapy (CRT) or for staging of prostate cancer [3–9]. There is a growing interest in DWI of baseline rectal tumors, especially for quantitative and functional techniques, aiming to identify tumors with favorable prognoses or to predict treatment response [10, 11]. For these techniques, image quality is crucial; however, little is known about the impact of micro-enemas on image artifacts when evaluating treatment-naïve rectal tumors.

Since 2011, we have applied a bisacodyl micro-enema, the standard micro-enema used at our department, before MRI of rectal cancers. Bisacodyl is a stimulative micro-enema that increases fluid secretion and motility of the large intestine [12]. We registered a third effect: the induction of submucosal edema. Recently, we published two studies demonstrating the benefits of using bisacodyl micro-enema before MRI of early rectal cancer ERC [13, 14]. We achieved diagnostic accuracy reaching levels comparable to endorectal ultrasound, and submucosal edema and the moderate amount of fluid in the rectum improved tumor delineation, reader confidence, and inter-reader agreement. As there is a shift toward early-stage cancers due to screening [15] and organ-saving treatment options are developing [16, 17], it is important to explore and utilize the possibilities of MRI in this patient population.

The objectives of this prospective experimental study were to evaluate the effects of a bisacodyl micro-enema as preparation before MRI of rectal tumors by assessing any change of submucosal edema in the healthy rectal wall, luminal fluid, rectal distension, and image quality and to evaluate patients’ acceptance.

## Materials and methods

### Ethics

This single-center study was approved by the local data protection officer and by the Regional Committee for Medical and Health Research Ethics South-East Norway (2015/1272). Written informed consent was obtained from all participants.

### Inclusion

From January 2016 to July 2017, we prospectively enrolled 73 consecutive patients to investigate the ability of MRI to identify ERC eligible for local excision after a rectal micro-enema. The final study population consisted of 50 patients who agreed to complete two MRI examinations, one without (MRex) and one with (MRin) bisacodyl micro-enema.

**Fig. 1:**
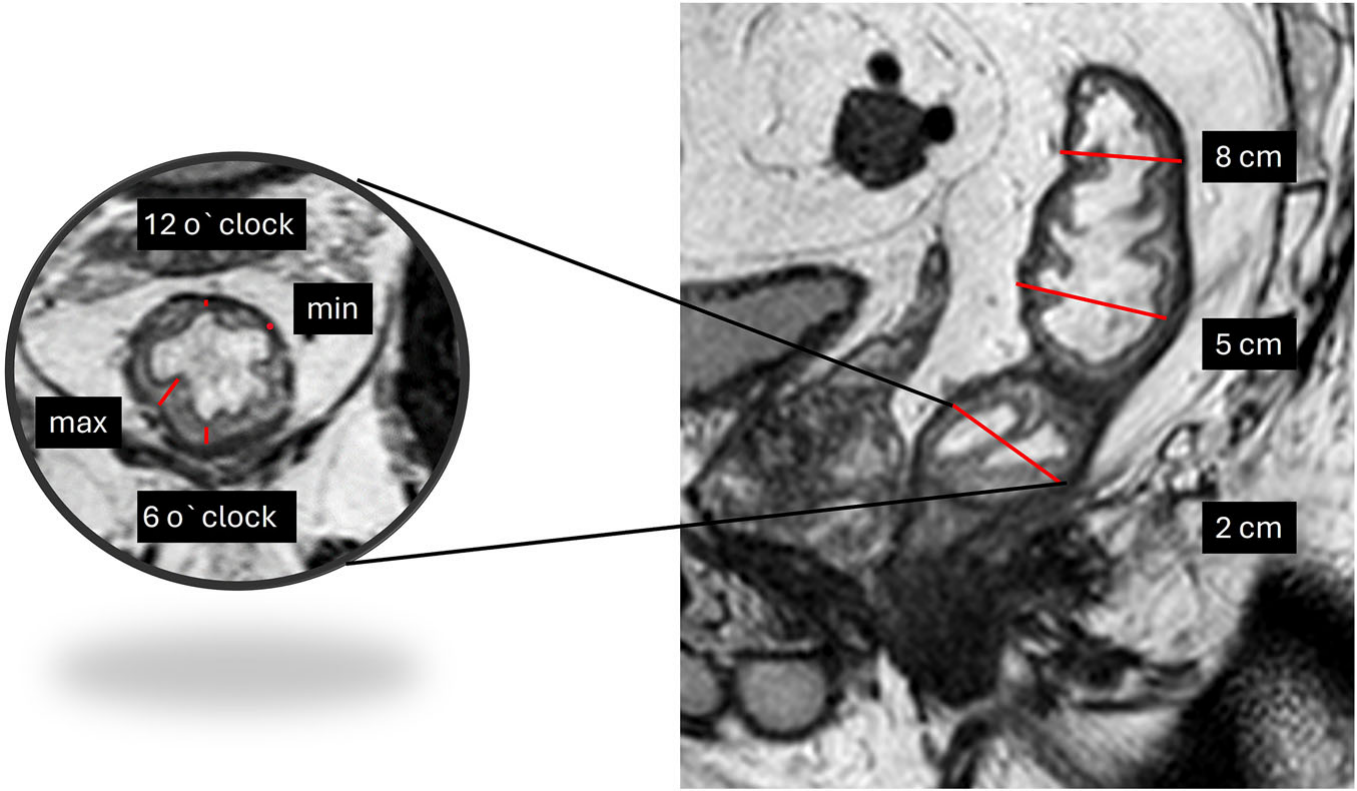
Sagittal and perpendicular T2W after micro-enema displaying levels (2, 5, and 8 cm orally from the anorectal junction) and location for submucosal measurements

Inclusion and exclusion criteria have been previously published [13] and are also provided in Electronic Supplementary Material (ESM) Fig. 1.

#### MRI technique

Three T2W sequences were performed: two 2D T2W high-resolution angulated perpendicular and parallel to the tumor base and one axial 3D T2W sequence. In addition, two DW sequences were obtained: one high-resolution parallel to the tumor base and one axial full field of view. Complete imaging data are previously published [13] and, in addition, provided in the ESM Text 1 and ESM Table 1.

### Patient preparation

The patients underwent dietary restrictions prior to the MRex and MRin, including only clear liquids for the last 4 h before the examination. For the MRin, the patients administered the bisacodyl micro-enema (Toilax Micro Enema; Orion Corp., Orion Pharma) (10 mg/5 mL) themselves in the radiology department 1 h before the examination. If not contraindicated, all patients received 1 mg of glucagon (Glucagon®, Novo Nordisk) IM right ahead of the examination and 20 mg of butylscopolamine (Buscopan®; Sanofi-Aventis) IV during the examination to reduce bowel movement artifacts. When required, a second dose of 20 mg of butylscopolamine was administered.

### Image analysis

Two radiologists with 20 (E.V., R1) and 15 (B.H., R2) years of experience in abdominal MRI independently interpreted the anonymized study MR images on a multimodality reading platform (Syngovia VB30®, Siemens Healthineers). Using the 3D T2 dataset, multiplanar reformats could be viewed in any plane [13].

### Submucosal edema, luminal fluid, and rectal distension

Submucosal edema, luminal fluid, and rectal distension were assessed by Reader1. Submucosal width was measured at four predefined locations (minimum and maximum width and width at 6 and 12 o’clock) at three levels, 2, 5, and 8 cm, orally to the ARJ (Fig. 1). Anal–oral extension of submucosal edema was registered on the sagittal images by measuring the longitudinal extension of increased signal intensity in the submucosa. Determining the volume of luminal fluid is difficult. Instead, we registered the anal-oral fluid extension. To evaluate rectal distension, the anterior-posterior diameter in the mid-rectum was measured, avoiding the tumor level.

### Image artifacts

To register the severity of the image artifacts, we used a figurative visual analog scale (fVAS), an exactly 10 cm line with absolute minimum and maximum scores at the boundaries and two reference points at 3.3 and 6.6 cm (ESM Fig. 2) with predefined definitions for all reference points with 0 = severe artifacts and 10 = no artifacts [18]. Clinically relevant artifacts were defined as a score < 5. The scores were recorded with a 10 cm ruler.

Both readers assessed blurring and motion artifacts on all three T2W sequences and distortion or hot spots on DWI high b-values in the axial and angulated plans. The artifacts were classified into four classes to evaluate artifact severity. Severe artifacts 0– <2.5: Not possible to assess details in the tumor and bowel wall. Moderate artifacts 2.5– <5: Assessment of details in tumor and bowel wall reduced. Mild artifacts 5–7.5: Detailed tumor and bowel wall assessment possible. Mild-no artifacts >7.5–10: Excellent visualization of tumor and bowel wall details. For both motion and susceptibility, the readers focused on artifacts of the tumor and bowel wall in the tumor height.

### Patient acceptance and side effects

To measure patient acceptance, the patients were asked to grade anal discomfort, abdominal pain, nausea, and rectal bleeding and rate the frequency of bowel movements by filling in questionnaires covering three different time points (ESM Figs. 3–5). The baseline questionnaire covered the last week before the first examination, and the MRex and MRin questionnaires, the first 24 h after the MRex and the MRin, respectively. The questionnaires were based on similar studies [19–21] and presented as 11-point Numerical Rating Scales (NRS), with 0 representing no symptoms or pain and 10 representing a very severe grade of symptoms or pain [19–22]. We adjusted a descriptive 4-point Verbal Rating Scale (VRS) for bowel movement and a 5-point VRS for rectal bleeding. The questionnaires were identical on all three time points; besides, in the MRin questionnaire, the patients also reported difficulties in the self-administration of the enema and immediate bleeding linked to the application. The grading of bleeding at application differed slightly from the other time points and was evaluated separately. In the MRex and MRin questionnaires, the patients graded the total experience of the MRI examination. Problems with self-application and reasons for discomfort could be registered in free text.

The side effects were validated by differences in mean NRS score and changes in clinically relevant side effects. A change in the NRS of 20% or 2 points between two measurements differing in time was considered clinically significant [22]. A score of 0 on the NRS was interpreted as no, 1–3 as mild, 4–6 as moderate, 7–9 as severe, and 10 as very severe symptoms or pain. For the VRS with 4- or 5-point scales, we defined a change of 1 point as clinically significant.

### Statistical analyses

All statistical analyses were performed using Stata (Statistical Software: Release 16. StataCorp LLC). According to the data distribution, a *t*-test, Wilcoxon signed-rank test, or McNemar’s test for paired samples was conducted. Inter-reader agreement for clinically relevant artifacts on MRex and MRin was calculated for motion and susceptibility artifacts. We found high agreement and prevalence for single parameters measuring image artifacts, and to avoid the paradox of Cohen’s Kappa, we used proportions of agreement and Gwet’s agreement coefficient 1 (AC1) [23, 24]. The estimated inter-reader agreement coefficients were interpreted using the Landis and Koch scale [25]: Gwet’s AC1 < 0 = poor, 0–0.20 = slight, 0.21–0.40 = fair, 0.41–0.60 = moderate, 0.61–0.80 = substantial, and 0.81–1.00 = almost perfect agreement.

## Results

In total, 50 patients underwent MRex and MRin. The inclusion flowchart, previously published [14], is provided in ESM Fig. 1. Patient and tumor characteristics are shown in Table 1.

**Table 1:** Patient and tumor characteristics

Variable	*n* (%)//median (iqr) (range)
Age	65 (14) (46–90)
Sex	
Men	26 (52)
Women	24 (48)
Histopathology	
Tis	32 (64)
T1	6 (12)
T2	9 (18)
T3	3 (6)
Tumor length:	
Benign (Tis), cm	35 (16) (15–120)
Malignant (> Tis), cm	41 (23) (20–68)
Location tumor:	
Low rectum, 0–5 cm from anal verge	17 (34)
Mid-rectum, > 5–10 cm from anal verge	28 (56)
Upper rectum, > 10–15 cm from anal verge	5 (10)
Antiperistaltic medication	
MRex Glucagon IM 1 mg	44 (88)
MRin Glucagon IM 1 mg	45 (90)
MRex Butylscopolamin IV 20 mg	47 (94)
MRin Butylscopolamin IV 20 mg	48 (96)
MRex Butylscopolamin IV, second dose 20 mg	15 (30)
MRin Butylscopolamin IV, second dose 20 mg	21 (42)
MRI examination	
Time interval between MRex and MRin, days	18 (11) (2–41)

*iqr* inter quartile range*, Tis* tumor in situ*, MRex* MRI without micro-enema, *MRin* MRI with micro-enema

### Antiperistaltic medication

There was no significant difference between MRex and MRin in the number of patients receiving glucagon IM or butylscopolamine IV before or during the MRI examination. A few more patients received a second dose of 20 mg of butylscopolamine during MRin. However, this difference did not reach statistical significance, with *p* = 0.17 (Table 1). Adherence to dietary restrictions was not registered.

### Submucosal edema, luminal fluid, and rectal distension

**Fig. 2:**
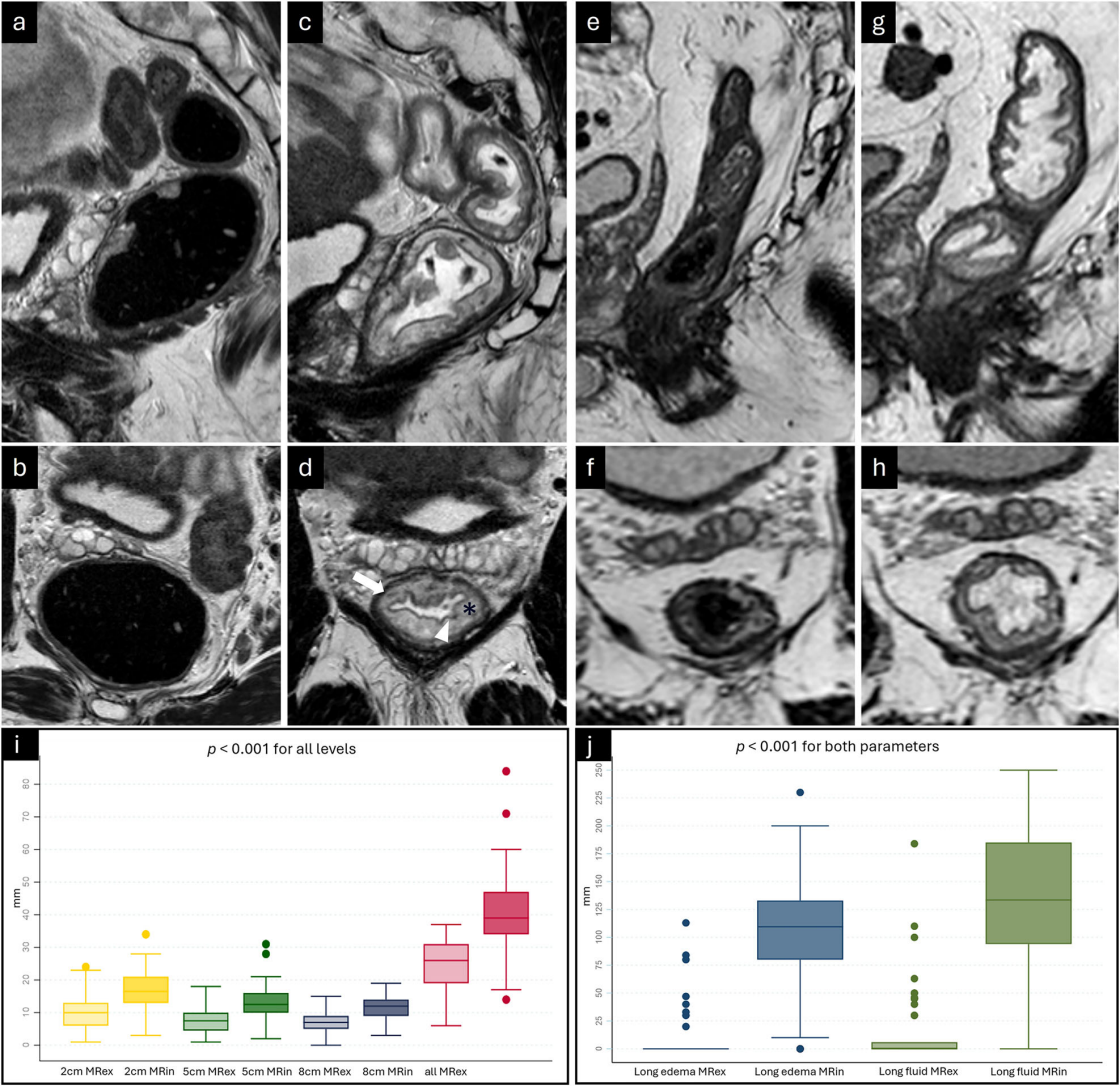
**a**–**h** T2 TSE of two patients, sagittal (upper row) and perpendicular (lower row). **a**, **b** Patient 1 without micro-enema; rectum distended due to copious feces. **c**, **d** A clean rectum after the micro-enema (white arrow, muscularis propria; black asterix, submucosa; white arrowhead, mucosa). **e**, **f** Patient 2 without micro-enema, deflated nearly empty rectum. **g**, **h** A clean rectum after the micro-enema. In both patients, submucosal edema and luminal fluid increased after the micro-enema. In patient 1, the micro-enema reduced the rectal diameter, and in patient 2, the rectum became marginally more distended due to fluid secretion. In patient 2, the submucosal width was only slightly increased, but the submucosal edema and luminal fluid enhanced the intramural contrast and the visibility of the layers. **i** Boxplot showing submucosal width measured at four predefined locations (minimum and maximum width and width at 6 and 12 o’clock) at three levels, 2, 5, and 8 cm orally to the anorectal junction. Boxplots are given for MRex and MRin at all levels and summarized across all levels. **j** Boxplot showing longitudinal extension of submucosal edema and luminal fluid. MRex, MRI without micro-enema; MRin, MRI with micro-enema; all MRexMRin, summed diameters of min, max, 6 o’clock, and 12 o’clock

The micro-enema induced a significant increase in the summed submucosal width for each level orally to the ARJ, separately and for the sum across all levels (Figs. 1, 2). Median width (range) 2 cm orally the ARJ was 10 mm (1–24) at MRex and 17 mm (3–34) at MRin, 5 cm orally the ARJ 8 mm (1–18) at MRex and 13 mm (2–31) at MRin, and at 8 cm orally the ARJ 7 mm (0–15) at MRex and 12 mm (3–19) at MRin.

On MRin, there was a significant increase in the extension of the submucosal edema from a median (range) of 0 mm (0–113) at MRex to 110 mm (0–230) at MRin (Fig. 2). The edema reached the mid-rectum in 8% (4/50) and the upper rectum or lower sigmoid in 82% (41/50) of the patients. In 10% (5/50) of the patients, no edema appeared. On MRex, only one patient, 2% (1/50), had well-expressed submucosal edema, whereas 14% (7/50) had sparse submucosal edema.

We also found a significant increase in anal-oral extensions of luminal fluid (Fig. 2), increasing from a median (range) of 0 mm (0–184) at MRex to 134 mm (0–250) at MRin. The rectal distension did not increase; mean anterior-posterior diameter measured 26 ± 9 mm at MRex and 26 ± 6 mm at MRin, *p* = 0.53. In 90% (45/50) of the cases, fluid reached the tumor level on MRin. On MRex, intraluminal fluid was registered in 28% (14/50) of the patients, always surrounding the tumor.

### Image artifacts

In T2 sequences, the readers found no significant difference in the frequency of clinically relevant motion artifacts between MRex and MRin (Table 2).

**Table 2:** Clinically relevant motion artifacts on T2W and susceptibility artifacts on DWI

	MRex		MRin		
	Clinically relevant *n* (%)	No-mild *n* (%)	Clinically relevant *n* (%)	No-mild *n* (%)	*p*-value
2D T2W perpendicular, Reader1	1 (2)	49 (98)	3 (6)	47 (94)	0.63
2D T2W parallel, Reader1	3 (6)	47 (94)	3 (6)	47 (94)	1.0
3D T2W axial, Reader1	0 (0)	50 (100)	2 (4)	48 (96)	0.5
2D T2W perpendicular, Reader2	0 (0)	50 (100)	0 (0)	50 (100)	1.0
2D T2W parallel, Reader2	0 (0)	50 (100)	0 (0)	50 (100)	1.0
3D T2W axial, Reader2	0 (0)	50 (100)	0 (0)	50 (100)	1.0
DWI axial, Reader1	8 (16)	42 (84)	1 (2)	49 (98)	0.04
DWI parallel, Reader1	25 (50)	25 (50)	4 (8)	46 (92)	< 0.001
DWI axial, Reader2	10 (20)	40 (80)	2 (4)	48 (96)	0.02
DWI parallel, Reader2	11 (22)	39 (78)	2 (4)	48 (96)	0.004

*T2W* T2 weighted images, *DWI* diffusion weighted images, *MRex* MRI without micro-enema, *MRin* MRI with micro-enema

**Fig. 3:**
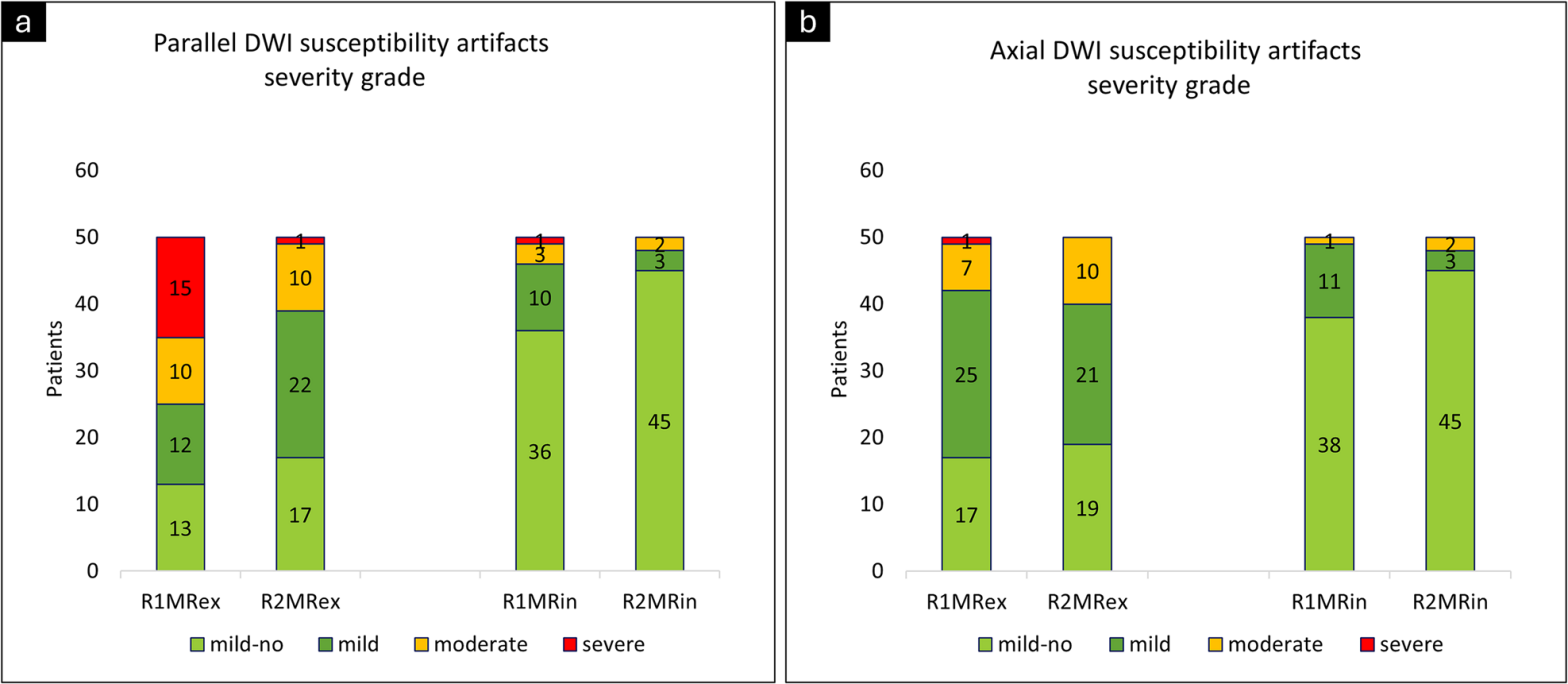
Severity grading of susceptibility artifacts, (**a**) axial plane, and (**b**) parallel plane. R1 Reader1, R2 Reader2, MRex MRI without micro-enema, MRin MRI with micro-enema

**Fig. 4:**
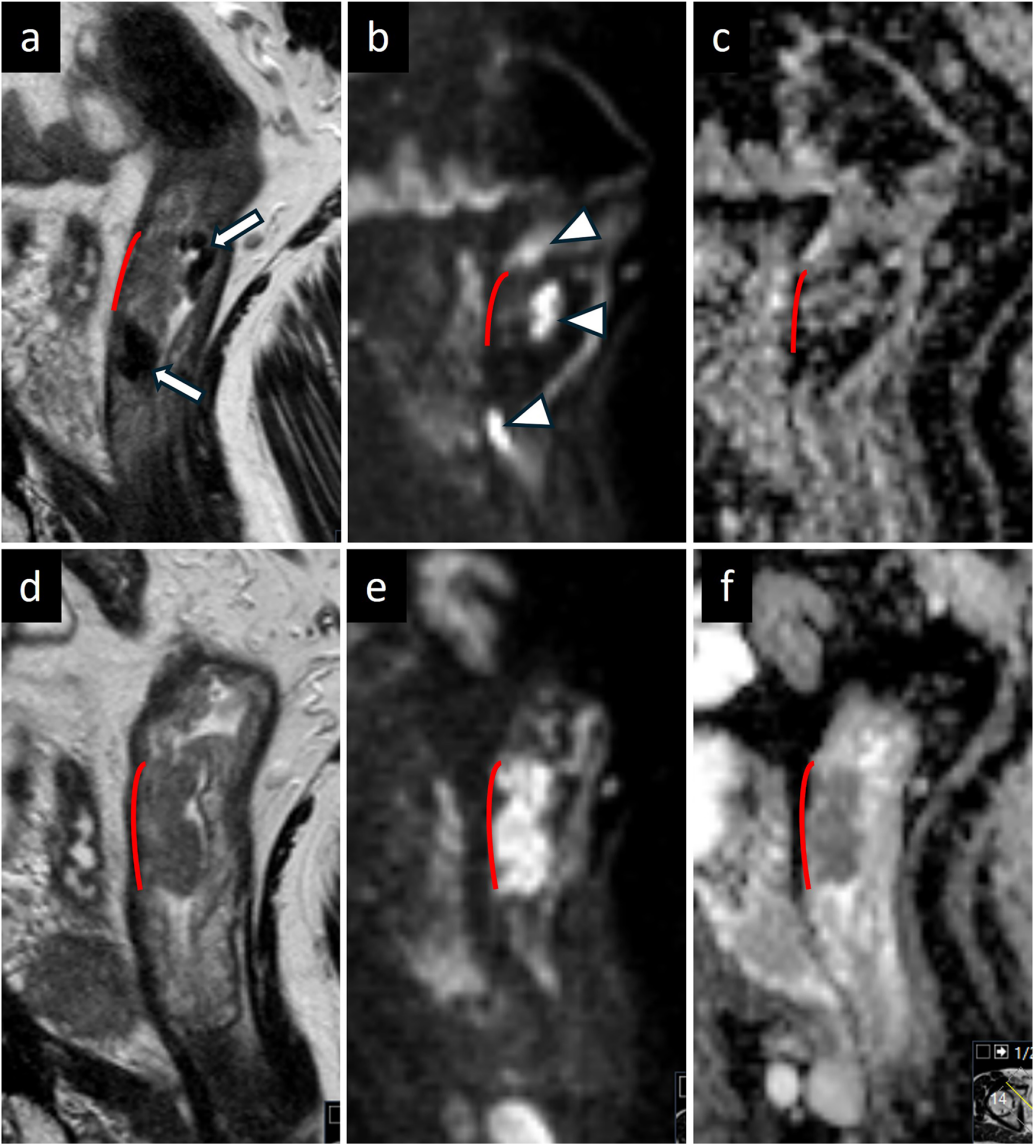
Sagittal plane MRI of a 3 cm long tumor at the anterior rectal wall, (**a**–**c**) without rectal micro-enema, and (**d**–**f**) with micro-enema. **a** T2 TSE, it is difficult to delineate the oral border of the tumor, and the tumor is partly surrounded by feces and gas. **b** DWI b-1000, difficult to identify the tumor, which is surrounded by hot spots. **c** ADC, difficult to identify the tumor. After the micro-enema, the tumor is well displayed on (**d**) T2 TSE, (**e**) DWI b-1000, and (**f**) ADC. Red line, tumor base; white arrow, feces or gas; white arrow heads, hot spots

Both readers observed significantly fewer susceptibility artifacts on MRin compared to MRex in both imaging planes (*p* < 0.04). Additionally, the severity of these artifacts decreased from MRex to MRin (*p* < 0.0001) for both readers (Table 2, Figs. 3, 4).

### Inter-reader agreement

The inter-reader agreement for motion artifacts being clinically relevant or not was almost identical for MRex and MRin and ranged from 94 to 100% in all three T2W sequences, with Gwet’s AC1 coefficient from 0.94 to 1.00. Inter-reader agreement for susceptibility artifacts being clinically relevant or not increased from MRex to MRin from 76% to 94% in the axial plane and from 64% to 88% in the parallel plane. Likewise, Gwet’s AC1 coefficient increased from MRex to MRin from 0.66 to 0.94 in the axial plane and from 0.33 to 0.86 in the parallel plane.

### Patient acceptance and side effects

**Fig. 5:**
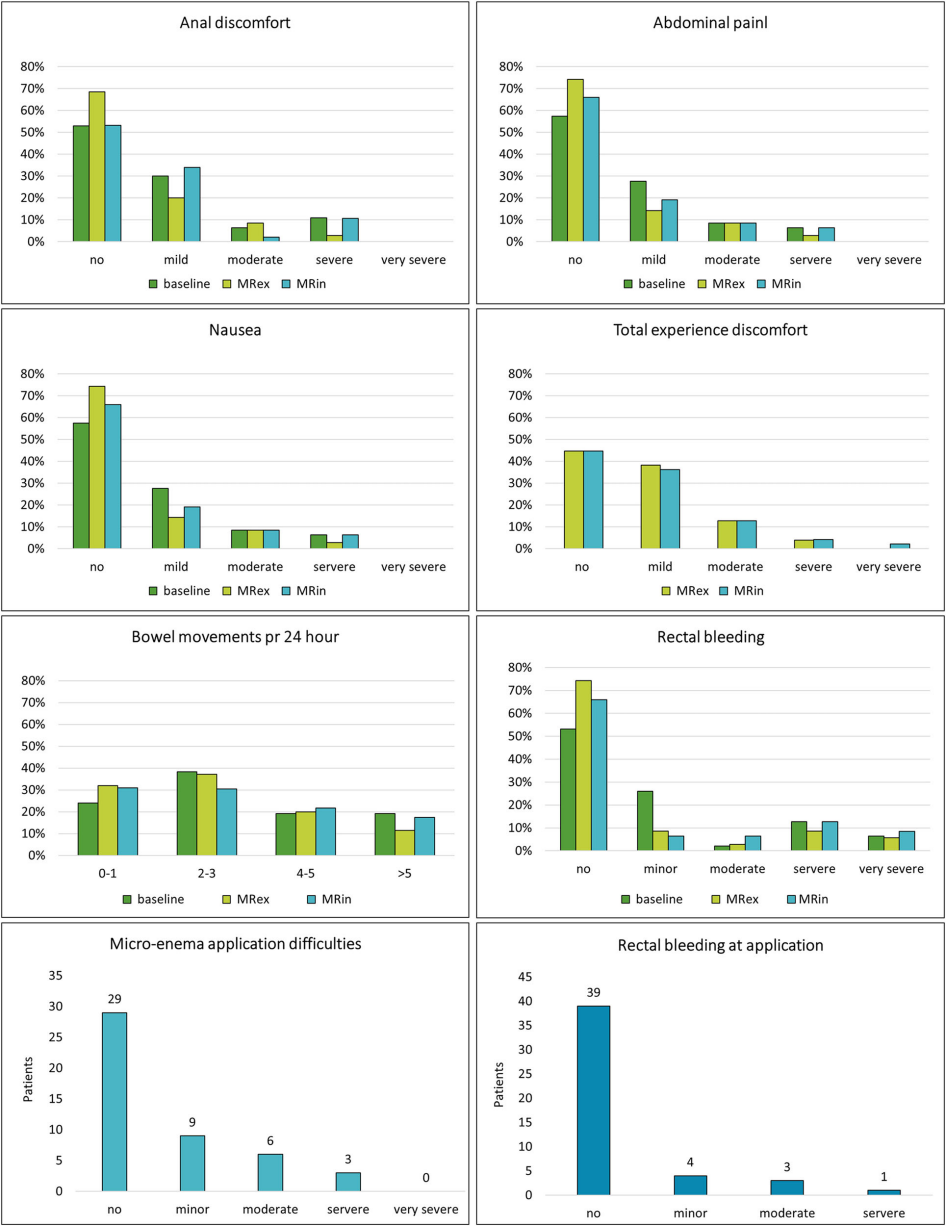
Proportion of patients experiencing side effects at baseline (last week before the first MRI) and in the first 24 h after MRex and MRin. The grading of bleeding at application differed slightly from the other time points, as it included only four options and is displayed separately. MRex, MRI without micro-enema; MRin, MRI with micro-enema; MRappl, MRI at application

Three of the 50 patients undergoing MRex and MRin did not answer the questionnaires for logistical reasons. The MRex questionnaire was completed by 35 patients only, as 12 started preparations for the next day’s surgery immediately after the MRex and only provided feedback regarding their overall experience. Most patients described no or only mild anal discomfort, rectal bleeding, nausea, or abdominal pain at all three time points (Tables 3, 4, and Fig. 5). The patients reported more nausea at MRin than MRex, but this did not reach clinical significance as the mean change was less than 2 points on the NRS scale. Rectal bleeding immediately following the application of the micro-enema was noted by 17% (8/47) of the patients with a mean NRS score of 1.3 ± 0.7, lower than at all other time points. Only one patient registered bleeding exclusively at application. All bleedings were self-limiting. The total number of bleedings at application did not differ from MRex and was significantly less than at baseline (*p* < 0.001) or MRin (*p* = 0.04).

**Table 3:** Side effects scored by numerical rating scale (NRS), 0 = no symptoms/not unpleasant and 10 = very severe symptoms/very unpleasant

	NRS score mean (SD, 95% CI)	Δ NRS score vs baseline mean (SD, 95% CI)^a^	vs baseline *p*-value	Δ NRS score MRex vs MRin mean (SD, 95% CI)^a^	MRex vs MRin *p*-value	*n* tot
Problems with ME application	1.4 (2.4, 0.7–2.1)	na	na	na	na	47
Anal discomfort baseline	1.8 (2.4, 1.1–2.5)	na	na	na	na	47
Anal discomfort MRex	0.9 (1.8, 0.3–1.6)	−0.9 (2.5, −1.8 to 0.06)	0.06	−0.5 (2.1, −1.2 to 0.3)	0.06	35^b^
Anal discomfort MRin	1.5 (2.3, 0.8–2.2)	−0.3 (2.5, −1.0 to 0.5)	0.5			47
Abdominal pain baseline	1.5 (2.2, 0.9–2.2)	na	na	na	na	47
Abdominal pain MRex	1.0 (1.9, 0.3–1.6)	−0.8 (2.4, −1.6 to 0.5)	0.01	−0.3 (1.7, −0.9 to 0.3)	0.3	35^b^
Abdominal pain MRin	1.2 (2.1, 0.6–1.8)	−0.3 (1.7, −0.8 to 0.2)	0.08			47
Nausea baseline	0.7 (1.3, 0.4–1.1)	na	na	na	na	47
Nausea MRex	0.6 (1.3, 0.2–1.1)	−0.3 (1.3, −0.7 to 0.2)	0.2	−0.7 (1.7, −1.2 to −0.07)	0.02	35^b^
Nausea MRin	1.1 (2.0, 0.5–1.7)	0.3 (1.8, −0.2 to 0.9)	0.68			47
Total experience MRex, discomfort^c^	1.6 (2.0, 1.0–2.1)	na	na	0.2 (2.1, −0.4 to 0.8)	0.96	47
Total experience MRin, discomfort^c^	1.8 (2.4, 1.1–2.5)					47

*ME appl* micro-enema application, *MRex* MRI without micro-enema, *MRin* MRI with micro-enema, *n* number, *n tot* total number, *Tot exp* total experience, *SD* standard deviation, *na* not applicable, *vs* versus, *CI* confidence interval

^a^ Negative mean values indicate symptoms less severe than the reference point

^b^12 patients direct to surgery after MRex, only filled in total experience in MRex form

^c^ Total experience not applicable for baseline, instead compared MRex versus MRin

**Table 4:** Rectal bleeding scored by verbal rating scale (VRS), 1 = no bleeding and 5 = very severe bleeding

Rectal bleeding	VRS score mean (SD, 95% CI)	Δ VRS score vs baseline mean (SD, 95% CI)^a^	vs baseline *p*-value	Δ VRS score MRex vs MRin mean (SD, 95% CI)^a^	MRex vs MRin *p*-value	*n* tot
Baseline	1.9 (1.3, 1.6–2.3)	na	na	na	na	47
MRex	1.6 (1.2, 1.2–2.1)	−0.2 (0.8, −0.5 to 0.06)	0.11	−0.1 (1.0, −0.4 to 0.3)	0.9	35^b^
MRin	1.9 (1.4, 1.5–2.3)	0 (0.9, −0.3 to 0.2)	0.57			47

*MRex* MRI without micro-enema, *MRin* MRI with micro-enema, *n* number, *n tot* total number, *SD* standard deviation, *na* not applicable, *vs* versus, *CI* confidence interval

^a^ Negative mean values indicate symptoms less severe than the reference point

^b^ 12 patients direct to surgery after MRex, only filled in total experience in MRex form

The patients reported no increase in bowel movements after MRin.

The most frequent problem reported by 28% (13/47) was technical issues concerning the application of the enema, like difficulties emptying the tube, no lubricant, or a sharp tube tip. Fear of injuring the tumor or hemorrhoids was expressed by 9% (4/47) of the patients. Side effects of the antiperistaltic medication, such as heartbeat and dry mouth, were mentioned by 6% (3/47), and 4% (2/47) missed a suitable restroom for the micro-enema application. The total experience of the MRI examination did not differ between MRex and MRin, and most patients reported no or only minor discomfort. Unpleasant experiences were reported by 26% (12/47) for both MRex and MRin, mainly linked to claustrophobia or the MRI examination itself as too noisy or too long.

## Discussion

Bisacodyl micro-enema used as a preparation before MRI of treatment-naïve rectal tumors induced a significant widening of the submucosa in the healthy rectal wall and increased the anal-oral extension of intraluminal fluid in the rectum and lower sigmoid without increasing rectal distension. The micro-enema reduced the incidence and severity of susceptibility artifacts on DWI, and there was no increase in motion artifacts on T2W images. Patient acceptance remained unchanged.

The submucosal edema occurred in most patients and was significantly larger at 2 cm, probably due to higher exposure to the enema in the lower rectum, where the fluid was delivered into the lumen. On MRin, 10% of the patients did not exhibit any submucosal edema. This might be due to technical problems with the application of the micro-enema; however, this was reported only by two of these patients in the self-reporting questionnaires. The induction of submucosal edema by bisacodyl micro-enema was previously described by Ciggaar et al using MRI to explore deep infiltrating endometriosis [26]. Studies investigating rectal tumors using non-stimulative micro-enemas acting through lubrication, softening, or osmosis did not report submucosal edema [7–9], nor did Kim et al [27] using bisacodyl suppositories 3 h before the MRI examination. Still, it is unclear whether this is due to missing edema or a lack of awareness.

There has been a concern that distention of the rectum may compress the mesorectum and reduce the distance between the tumor and the mesorectal fascia (MRF), leading to overstaging and overtreatment [28]. Later studies, however, indicate that this is not the case at the tumor level, and compression of the mesorectum due to rectal distension probably mainly occurs in the healthy rectal wall [29, 30]. Despite a significant increase in luminal fluid after bisacodyl micro-enema, we found no significant increase in rectal distension, neither in extra-tumoral segments nor, as we previously have published, at the tumor level [14]. The fluid secretion following a bisacodyl micro-enema appears moderate and insufficient to distend the rectum.

Rectal filling using water or gel can increase tumor visibility, especially for small tumors and tumors close to the ARJ. However, it also implies some disadvantages, such as increasing motion artifacts, stretching and thinning of the rectal wall, and overestimation of the distance between the tumor and ARJ [29, 31, 32]. The effect on T-staging is controversial. Some studies have found a slight improvement [29, 31], while increased overstaging with rectal filling, especially for early-stage tumors, was reported by another study using gel [30]. The challenges with rectal filling seem related to the distention and overstretching of the rectum, and filling with not more than 30 mL has been suggested [2]. Some luminal filling may be advantageous and potentially improve tumor visualization, T-stage accuracy, and reader confidence [14, 29, 31]. Bisacodyl micro-enema, as a secretory laxative, combines the beneficial effects of rectal cleansing and simultaneously moderately fluid filling of the rectum.

**Fig. 6:**
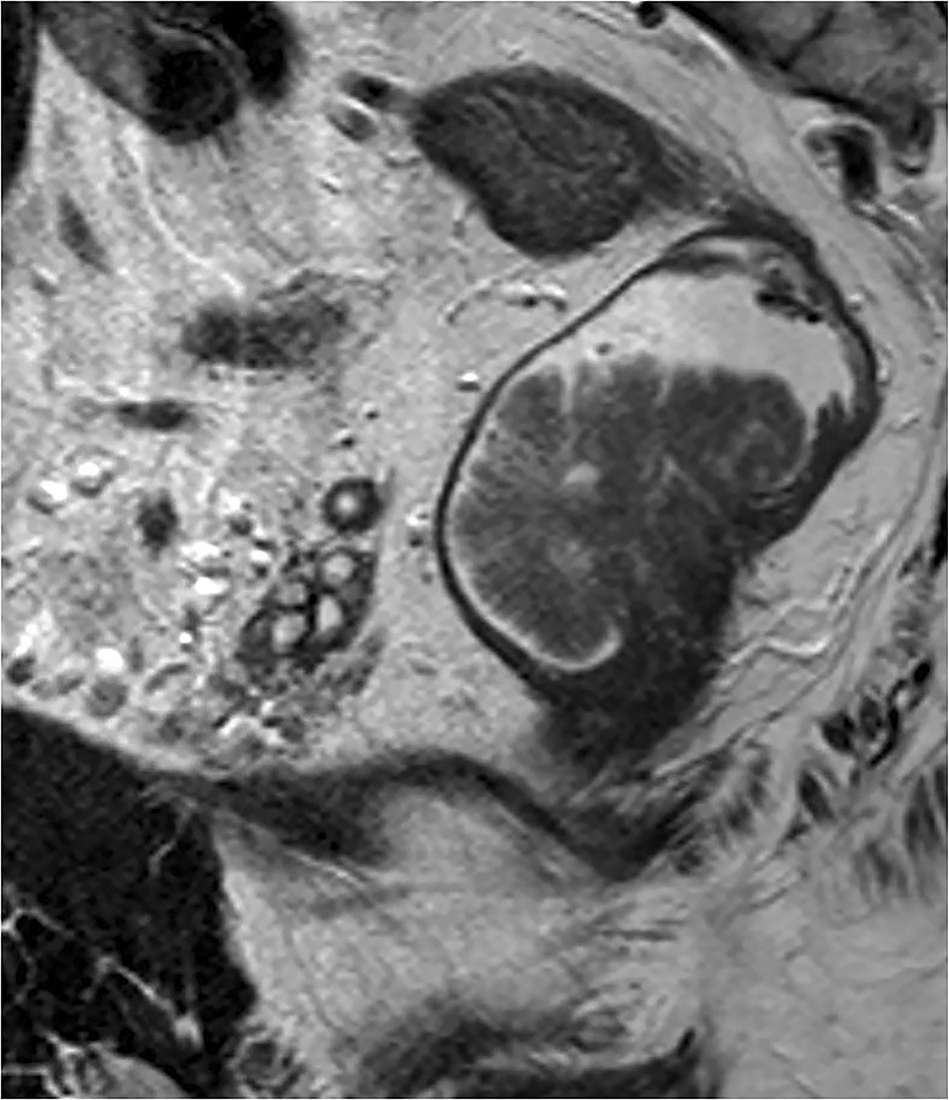
Sagittal T2WI without micro-enema showing a sessile tumor with tumor base at the posterior rectal wall. Tumor is surrounded by fluid, probably mucus produced by the tumor

On MRex, luminal fluid was registered in only 28% of the patients and was always located close to the tumor, probably representing mucus produced by the tumor (Fig. 6).

Regarding ERC, tumor invasion into the thin layers of the rectal wall is mainly assessed on T2W images, a sequence prone to motion artifacts; therefore, all patients underwent dietary restrictions and administration of two different spasmolytic agents. Several studies investigating prostate cancer have found no increase in motion artifacts following micro-enemas [3, 4, 6]. However, these studies investigated mainly the motion artifacts affecting the prostate, and the results may not fully apply to rectal tumors. In addition, the micro-enemas used were non-stimulative. As bisacodyl micro-enema directly stimulates peristalsis, registering the impact on motion artifacts was of special interest. However, we found no increase in clinically relevant motion artifacts in any of the three T2W sequences after the bisacodyl micro-enema. This is in line with Ciggaar et al [26] studying MRI of endometriosis after a bisacodyl micro-enema.

For response evaluation of rectal cancer after CRT, MRI with DWI is recommended [1, 2]. DWI is sensitive to luminal gas, and clinically relevant susceptibility artifacts are shown in up to 24% of DWI of rectal cancer post-CRT [7]. This may be overcome by rectal micro-enemas or rectal filling with gel, as both have been found to reduce susceptibility artifacts [7–9]. In our study, the readers found a significant reduction in the incidence and severity of susceptibility artifacts in both DWI planes after the micro-enema, a finding partly in line with previous studies using non-stimulative micro-enemas [7–9]. Griethuysen et al only investigated tumors achieving complete response post-CRT, and Jayaprakasam et al found reduced susceptibility artifacts only on post-total neoadjuvant treatment and not on treatment-naïve baseline tumors. The missing effect on baseline images was explained by a limited gaseous dilatation and mobility of the bowel because of a significant intraluminal tumor. The tumor size may explain our study’s advantageous effect on susceptibility artifacts, as we investigated ERC and tumors that were less voluminous.

Micro-enemas, as preparation before MRI of rectal tumors, are supposed to be harmless for the patients [3, 7]. However, besides the fear of adverse effects on image quality, our main concern was the possibility of injuries to the tumor, inducing bleeding and pain. Common side effects reported to Drugs.com include abdominal cramps, abdominal pain, diarrhea, nausea, and local anorectal burning and discomfort [33]. Rarely, colitis, allergic, or anaphylactoid reactions are registered. In our study, most patients had no or mild symptoms at baseline, and there was no clinically relevant difference when comparing MRex and MRin. Bleeding at the micro-enema application was reported by eight patients. As only one bleeding was rated as severe in the self-reporting form, and all bleedings were self-limiting, we consider using a bisacodyl micro-enema as preparation before MRI of ERC as safe. Regarding the self-administration of the micro-enema, 19 patients reported minor or moderate inconvenience during the application or technical problems, mainly linked to the application of the micro-enema. Improving the design of the applicator tube and ensuring access to a suitable restroom could enhance the overall patient experience and acceptance.

Our study is not without limitations. We attempted to minimize the readers’ recall bias by assigning MRex and MRin of the same patient to two separate reading sessions with a minimum interval of 4 weeks. Still, the readers may have been biased in assessing image artifacts, as hiding the exposure to an enema was impossible. Due to logistical reasons, only 74% of the patients finished the MRex questionnaire because the MRex was too close to surgery. The smaller sample size may affect results, statistical power, and precision. However, the missing questionnaires were all after MRex, making it unlikely that this should lead to an underestimation of complications and side effects of the micro-enema.

Another potential limitation is the use of invalidated questionnaires. Nevertheless, NRS works well in pain assessment [34], and our questionnaires were based on studies with similar designs [19–21]. For the maximum score, we did not use the phrase “as bad as could be” but “very severe,” more in line with the EORTC QLQ-CR29 [35]. For bowel movements and rectal bleeding, we used VRS as we wanted a more figurative and quantitative grading, and the items in EORTC QLQ-CR29, QLQ-ANL27, or QLQ-C30 or Common Terminology Criteria for Adverse Events (CTCAE) [36] did not meet these requirements.

## Conclusion

In conclusion, bisacodyl micro-enema used as preparation before MRI of rectal tumors significantly increased the submucosal width and amount of intraluminal fluid without increasing the rectal distension. It improved image quality and was well tolerated by the patients.

## Supplementary information

ELECTRONIC SUPPLEMENTARY MATERIAL

## Abbreviations

ARJ: Anorectal junction
CRT: Chemoradiotherapy
DWI: Diffusion weighted imaging
EORTC QLQ-ANL27: The European Organization for Research and Treatment of Cancer quality of life questionnaire for anal cancer 27
EORTC QLQ-C30: The European Organization for Research and Treatment of Cancer core quality of life questionnaire 30
EORTC QLQ-CR29: The European Organization for Research and Treatment of Cancer quality of life questionnaire for colorectal cancer 29
ERC: Early rectal cancer
ESM: Electronic Supplementary Material
Gwet’s AC1: Gwet’s agreement coefficient 1
MRex: MRI without bisacodyl micro-enema
MRin: MRI with bisacodyl micro-enema
NRS: Numerical Rating Scale
SD: Standard deviation
VRS: Verbal Rating Scale
