# Supplementary material for: Bisacodyl micro-enema before MRI of rectal tumors: effects on rectum, image quality and patient acceptance
Source: Eur Radiol. 2025 Sep 17;36(3):1874–86. doi: 10.1007/s00330-025-11996-1 (PMC12963163; doi:10.1007/s00330-025-11996-1)
Supplement: Supplementary file 1 — ELECTRONIC SUPPLEMENTARY MATERIAL [file 330_2025_11996_MOESM1_ESM.docx]

## Electronic Supplementary Material ESM

**ESM1 Fig 1** inclusion flowchart


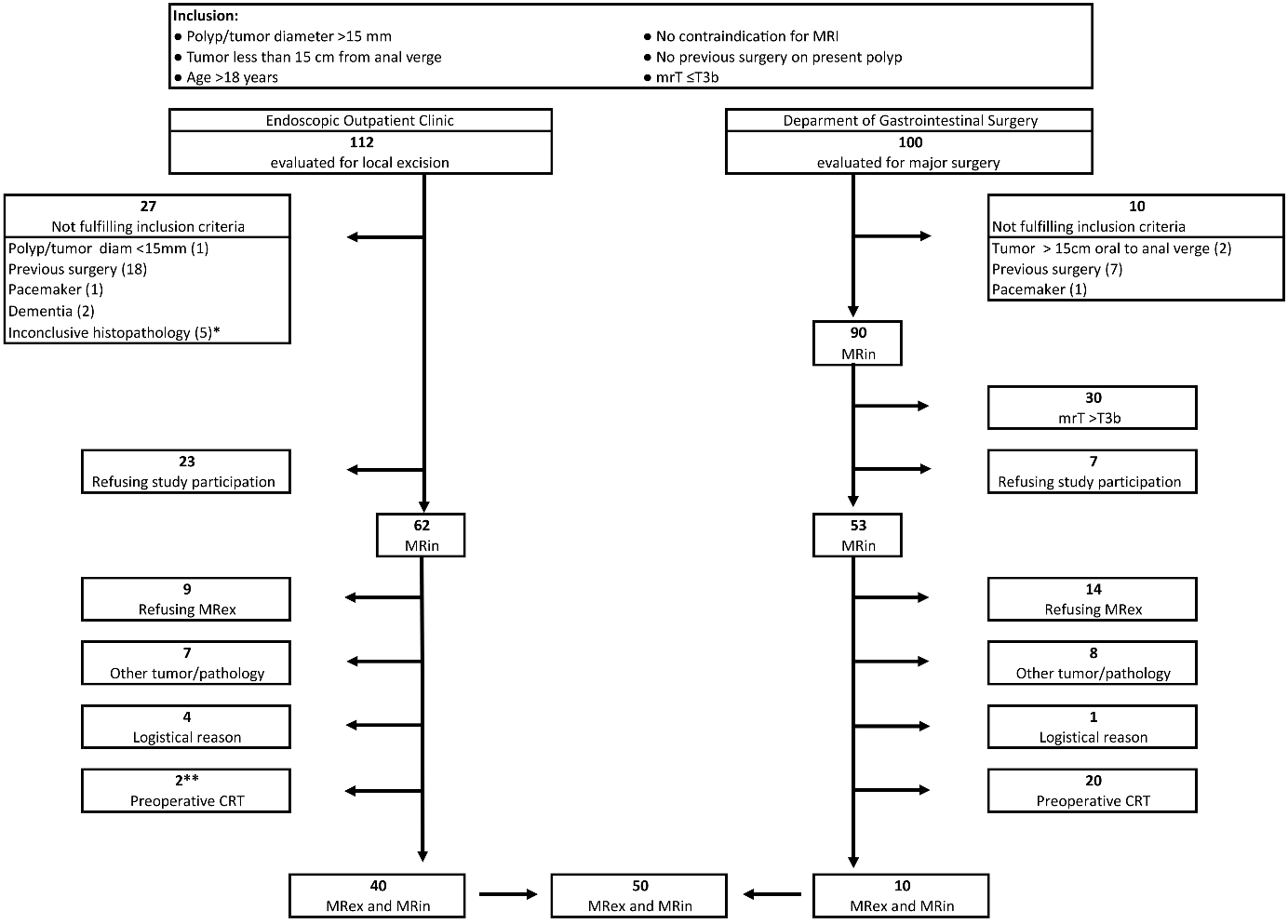


**ESM Fig. 1** Flowchart showing inclusion and exclusion. Consecutive evaluation of all patients referred to our institution for primary treatment of rectal tumors. *CRT* chemoradiotherapy, *MRin* MR with rectal micro enema, *MRex* MR without rectal micro enema.

**ESM text 1** MRI acquisition information

The MRI examinations were performed on a Philips Achieva 1.5 T (Philips Medical Systems) with a 32-channel cardiac coil or a Siemens Aera 1.5 T (Siemens Healthineers) with a body 30 coil. From the promontory to the anal verge, an axial 3D T2 weighted sequence (T2W) with sagittal and coronal reformats was acquired. The sequence was close to isotropic and allowed post-processing of multiplanar reformats in any direction. At tumor height, two high-resolution 2D T2W turbo spin-echo images were acquired, one perpendicular and one parallel to the long axis of the rectum and tumor. Diffusion weighted imaging was performed in two plans, one axial full field of view from the promontory to the anal verge and one high resolution angulated parallel to the long tumor axis, identical to the angulation plane of the high-resolution 2D T2W turbo spin-echo.

**ESM Table 1** MR imaging acquisition parameters for Philips Achieva 1.5 T and Siemens Aera 1.5T

| **1.5 Tesla (Phillips Achiva)** | **T2W TSE** | **T2W TSE** | **T2 3D VISTA**^a^ | **DWI** | **DWI** | **T1 3D VISTA** |  |
| --- | --- | --- | --- | --- | --- | --- | --- |
| Imaging planes | Oblique axial^b^ | Oblique parallel^c^ | Axial with sagittal and coronal reformates | Oblique parallel | Axial | Coronal |  |
|  |  |  |  |  |  |  |  |
| Repetition time/Echo time(msec) | 4695/90 | 4695/90 | 1175/85 | 1262/83 | 2808/69 | 250/14 |  |
| Bandwidth (Hz/pixel) | 195 | 195 | 445 | 2234 | 2629 | 430 |  |
| Field of view (mm) | 160 x 160 | 160 x 160 | 289/190 | 20 x 20 | 330 x 330 | 460 x 460 |  |
| Acquisition Matrix | 256/224 | 256/224 | 292/252 | 80 x 117 | 112 x 107 | 3841/382 |  |
| Section thickness/gap (mm) | 3/0.3 | 3/0.3 | 1/-0.5 | 5/1 | 5/1 | 1.2/-0.6 |  |
| Number of signals acquired/b-value | 6 | 6 | 1 | 8/b-0 | 3/b-0 | 2 |  |
|  |  |  |  | 8/b-500 | 3/b-500 |  |  |
|  |  |  |  | 8/b-1000 | 3/b-1000 |  |  |
|  |  |  |  |  |  |  |  |
| **1.5 Tesla (Siemens Aera)** | **T2W TSE** | **T2W TSE** | **T2 3D SPACE**^d^ | **DWI** | **DWI** | **T1 3D SPACE** |  |
| Imaging planes | Oblique axial | Oblique parallel | Axial with sagittal and coronal reformates | Oblique parallel | Axial | Coronal |  |
| Repetition time/Echo time(msec) | 4250/82 | 4250/82 | 1300/90 | 3400/69 | 3800/65 | 350/19 |  |
| Bandwidth (Hz/pixel) | 225 | 225 | 698 | 1445 | 1812 | 345 |  |
| Field of view (mm) | 200x200 | 200x200 | 256x256 | 200x200 | 368x248 | 384x384 |  |
| Acquisition Matrix | 320x320 | 320x320 | 256x256 | 128x128 | 184/124 | 384x384 |  |
|  |  |  |  |  |  |  |  |
| Section thickness/gap (mm) | 3/0.3 | 3/0.3 | 1.1/0 | 5/0 | 4/0.8 | 1.3/0 |  |
| Number of signals acquired/b-value | 3 | 2 | 1.4 | 3/b-50 | 4/b-500 | 1.4 |  |
|  |  |  |  | 4/b-300 | 9/b-1000 |  |  |
|  |  |  |  | 11/b-700 |  |  |  |
|  |  |  |  | Calculated b-1500 |  |  |  |

**ESM Table 1**

a VISTA Volumetric Isotropic TSE Acquisition = [3D fast spin echo](https://radiopaedia.org/articles/3d-fast-spin-echo-mri-sequence-1?lang=us): isotropic 3D sequences, allowing multiplanar reformats

b Oblique axial = Perpendicular to the long axis of rectum of tumor

c Oblique parallel = Parallel to the long axis of rectum and tumor

d SPACE = Sampling perfection with application-optimized contrasts using different flip angle evolution = [3D fast spin echo](https://radiopaedia.org/articles/3d-fast-spin-echo-mri-sequence-1?lang=us): isotropic 3D sequences, allowing multiplanar reformats

**ESM Fig 2**


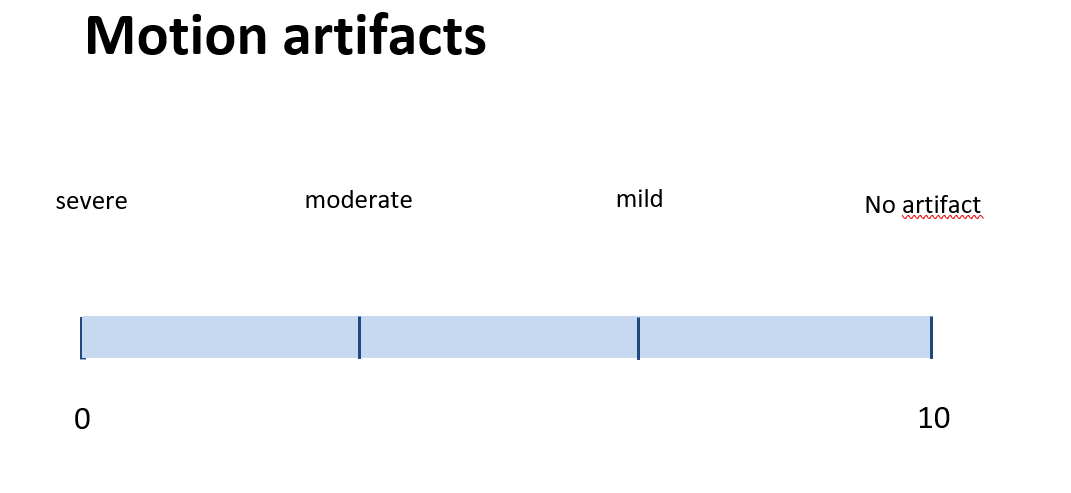


Example on figurative visual analogue scale (fVAS) for image artifacts with reference points at 0, 3.3, 6.6 and 10 cm.

**ESM Fig 3**

Questionnaire: **MRI baseline**

Please answer the questions before the first MRI examination to assess your symptoms the last week. Please encircle the best suitable alternative.

1. How often did you pass stool the last week before the MRI examination?
   1. 0-1 times a day
   2. 2-3 times a day
   3. 4-5 times a day
   4. > 5 times a day
2. Did you have anal bleedings the last week?
   1. No
   2. A little (sometimes stripes of blood on toilet paper or stool)
   3. Moderate (always stripes of blood on toilet paper or stool)
   4. Severe (water in toilet sometimes turning red)
   5. Very severe (water in toilet always turning red and blood clots )
3. Did you feel anal discomfort when not on toilet the last week? Please encircle the best suitable alternative on a scale from 0 = no discomfort to 10 = very severe discomfort.

0 1 2 3 4 5 6 7 8 9 10

no discomfort severe discomfort

1. Did you have abdominal pain the last week? Please encircle the best suitable alternative on a scale from 0 = no pain to 10 = very severe pain.

0 1 2 3 4 5 6 7 8 9 10

no pain very severe pain

1. Have you felt nauseated the last week?  Please encircle the best suitable alternative on a scale from 0 = no nausea to 10 = severe nausea.

0 1 2 3 4 5 6 7 8 9 10

no nausea severe nausea

**ESM Fig 4**

Questionnaire: **MRI baseline**

Please answers the questions 24 hours after the MRI examination by encircling the best suitable alternative.

1. How often did you pass stool in the first 24 hours after the MRI examination?
   1. 0-1 times
   2. 2-3 times
   3. 4-5 times
   4. > 5 times
2. Did you have anal bleedings the first 24 hours after the MRI examination?
   1. No
   2. A little (sometimes stripes of blood on toilet paper or stool)
   3. Moderate (always stripes of blood on toilet paper or stool)
   4. Severe (water in toilet sometimes turning red)
   5. Very severe (water in toilet always turning red and blood clots )
3. Did you feel anal discomfort when not on toilet the first 24 hours after the MRI examination? Please encircle the best suitable alternative on a scale from 0 = no discomfort to 10 = very severe discomfort.

0 1 2 3 4 5 6 7 8 9 10

no discomfort severe discomfort

1. Did you have abdominal pain in the first 24 hours after the MRI examination? Please encircle the best suitable alternative on a scale from 0 = no pain to 10 = very severe pain.

0 1 2 3 4 5 6 7 8 9 10

no pain very severe pain

1. Have you felt nauseated in the first 24 hours after the MRI examination?  Please encircle the best suitable alternative on a scale from 0 = no nausea to 10 = severe nausea.

0 1 2 3 4 5 6 7 8 9 10

no nausea severe nausea

1. How would you rate your overall experience of the MRI examination, including the micro-enema? Please encircle the best suitable alternative on a scale from 0 = not unpleasant to 10 = very unpleasant.

0 1 2 3 4 5 6 7 8 9 10

not unpleasant very unpleasant

1. If the total experience of the MRI examination was unpleasant, can you please elaborate?

**ESM Fig 5**

Questionnaire: **MRI baseline**

Please answer the questions 24 hours after the MRI examination by encircling the best suitable alternative.

1. Any problems with the application of the enema? Grade the problems on a scale from 0= no problems to 10 = severe problems.

0 1 2 3 4 5 6 7 8 9 10

no problem severe problems

If any problems occurred, can you please explain?

1. Did you have anal bleedings immediately after applying the enema?
   1. No
   2. A little (stripes of blood on toilet paper or stool)
   3. Moderate (water in toilet turning red)
   4. Severe (water in toilet turning red and blood clots)
2. How often did you pass stool in the first 24 hours after the MRI examination?
   1. 0-1 times
   2. 2-3 times
   3. 4-5 times
   4. > 5 times
3. Did you have anal bleedings the first 24 hours after the MRI examination?
   1. No
   2. A little (sometimes stripes of blood on toilet paper or stool)
   3. Moderate (always stripes of blood on toilet paper or stool)
   4. Severe (water in toilet sometimes turning red)
   5. Very severe (water in toilet always turning red and blood clots )
4. Did you feel anal discomfort when not on toilet the first 24 hours after the MRI examination? Please encircle the best suitable alternative on a scale from 0 = no discomfort to 10 = very severe discomfort.

0 1 2 3 4 5 6 7 8 9 10

no discomfort severe discomfort

1. Did you have abdominal pain in the first 24 hours after the MRI examination? Please encircle the best suitable alternative on a scale from 0 = no pain to 10 = very severe pain.

0 1 2 3 4 5 6 7 8 9 10

no pain very severe pain

1. Have you felt nauseated in the first 24 hours after the MRI examination?  Please encircle the best suitable alternative on a scale from 0 = no nausea to 10 = severe nausea.

0 1 2 3 4 5 6 7 8 9 10

no nausea severe nausea

1. How would you rate your overall experience of the MRI examination, including the micro-enema? Please encircle the best suitable alternative on a scale from 0 = not unpleasant to 10 = very unpleasant.

0 1 2 3 4 5 6 7 8 9 10

not unpleasant very unpleasant

1. If the total experience of the MRI examination was unpleasant, can you please elaborate?
